# Nobel Turing Challenge: creating the engine for scientific discovery

**DOI:** 10.1038/s41540-021-00189-3

**Published:** 2021-06-18

**Authors:** Hiroaki Kitano

**Affiliations:** The Systems Biology Institute, Tokyo, Japan; Okinawa Institute of Science and Technology Graduate School, Okinawa, Japan; Sony Computer Science Laboratories, Inc., Tokyo, Japan; Sony AI, Inc., Tokyo, Japan; and The Alan Turing Institute, London, UK

**Keywords:** Computational biology and bioinformatics

## Abstract

Scientific discovery has long been one of the central driving forces in our civilization. It uncovered the principles of the world we live in, and enabled us to invent new technologies reshaping our society, cure diseases, explore unknown new frontiers, and hopefully lead us to build a sustainable society. Accelerating the speed of scientific discovery is therefore one of the most important endeavors. This requires an in-depth understanding of not only the subject areas but also the nature of scientific discoveries themselves. In other words, the “science of science” needs to be established, and has to be implemented using artificial intelligence (AI) systems to be practically executable. At the same time, what may be implemented by “AI Scientists” may not resemble the scientific process conducted by human scientist. It may be an alternative form of science that will break the limitation of current scientific practice largely hampered by human cognitive limitation and sociological constraints. It could give rise to a human-AI hybrid form of science that shall bring systems biology and other sciences into the next stage. The Nobel Turing Challenge aims to develop a highly autonomous AI system that can perform top-level science, indistinguishable from the quality of that performed by the best human scientists, where some of the discoveries may be worthy of Nobel Prize level recognition and beyond.

## Nobel Turing Challenge as an ultimate grand challenge

Understanding, reformulating, and accelerating the process of scientific discovery is critical in solving problems we are facing and exploring the future. Building the machine to make it happen could be one of the most important contribution to society, and it will transform many areas of science and technology including systems biology. Since scientific research has been one of the most important activities that drove our civilization forward, the implications of such development will be profound.

Attempts to understand the process of scientific discoveries have a long tradition in the philosophy of science as well as artificial intelligence. Karl Popper introduced a concept of falsifiability as a criterion and process of solid scientific process but the process of hypothesis and concept generation do not have particular logic behind it^1^. Thomas Kuhn proposed a concept of a Paradigm shift where two competing paradigms are incommensurable and a set of knowledge, rather than a single knowledge, has to be switched with the transition of paradigm^2^. Imre Lakatos reconciles them by proposing actual science makes progress based on a “research program” composed of a hardcore that is immune to revision and flexible peripheral theories^3^. Contrary to these positions, Paul Feyerabend argued that there are no methodological rules in the scientific process^4^. Although these arguments are important thoughts in the philosophy of science, ideas are philosophical and not concrete to be implemented computationally. In addition, these studies are focused on how science is carried out as a part of human social activities. A rare example of implementing such concepts can be seen in the model inference system implemented by Ehud Shapiro that reflects Popper’s falsifiability^5^.

Not surprisingly, scientific discovery has been a major topic in artificial intelligence research that dates back to DENDRAL^6^ and META-DENDRAL, followed by MYCIN, BEACON^7^, AM, and EURISKO^6,8^. It continues to be one of the main topics of AI^9,10^. Recently, an automated experimental system that closed-the-loop of hypothesis generation, experimental planning, and execution has developed for budding yeast genetics that clearly marks the next step towards an AI Scientist^11–13^. While these pioneering works have focused on a single data set or a specific task using limited resources, it signifies the state-of-the-art of technology today that can be the basis of more ambitious challenges.

The obvious next step is to develop a system that makes scientific discoveries that shall truly impact the way we do science and aim for major discoveries. Therefore, I propose the launch of a grand challenge to develop AI systems that can make significant scientific discoveries that can outperform the best human scientist, with the ultimate purpose of creating the alternative form of scientific discovery^14^. Such a system, or systems, may be called “AI Scientist” that is most likely a constellation of software and hardware modules dynamically interacting to accomplish tasks. Since the critical feature that distinguishes it from conventional laboratory automation is its capability to generate hypotheses, learn from data and interactions with humans and other parts of the system, reasoning, and a high level of autonomous decision-making, the term “AI Scientist” best represents the characteristics of the system to be developed. The best way to accelerate the grand challenge of this nature is to define a clear mission statement with an audacious yet provocative goal such as winning the Nobel Prize^14^. Therefore, I propose the Nobel Turing Challenge as a grand challenge for artificial intelligence that aims at “developing AI Scientists capable of autonomously carrying out research to make major scientific discoveries and win a Nobel Prize by 2050”. While the previous article^14^ focused on rationales for such a challenge with emphasis on human cognitive limitations and needs for exhaustive search of hypothesis space, this article formulates the vision as the Nobel Turing Challenge and implications of massive and unbiased search of hypothesis space and verification, architectural issues, and interaction with human scientists are discussed as a transformative paradigm in science. The distinct characteristic of this challenge is to field the system into an open-ended domain to explore significant discoveries rather than rediscovering what we already know or trying to mimic speculated human thought processes. The vision is to reformulate scientific discovery itself and to create an alternative form of scientific discovery.

The accomplishment of this challenge requires two goals to be achieved that are: (1) to develop an AI Scientist that performs scientific research highly autonomously enabling scientific discoveries at scale, and (2) to develop an AI Scientist capable of making strategic choices on the topic of research, that can communicate in the form of publications and other means to explain the value, methods, reasoning behind the discovery, and their applications and social implications. When both goals are met, the machine will be (almost) comparable to the top-level human scientist as well as being scientific collaborators. The question and challenge is whether the machine will be indistinguishable from the top-level human scientists and most likely pass the Feigenbaum Test which is a variation of the Turing Test^15^, or whether it will exhibit patterns of scientific discovery that are different from human scientists.

It should be noted “winning the Nobel Prize” is used as a symbolic target illustrating the level of discoveries the challenge is aiming at. The value lies in the development of machines that can make discoveries continuously and autonomously, rather than winning any award including the Nobel Prize. It is used as a symbolism that triggers inspiration and controversy.

At the same time, the implication of the statement that explicitly aims to win the Nobel Prize poses a series of interesting questions whether such AI systems making a decisive discovery may also evolve to be indistinguishable from the top human scientist (passing the Feigenbaum Test^16^). As witness in case of Satoshi Nakamoto’s blockchain and bitcoin, there is a case where a decisive contribution was simply published as a blogpost^17^ and taken seriously, yet no one ever met him and his identity (at the time of writing) is a complete mystery. Given the possibility of creating a highly sophisticated virtual agent to interact with a human, with natural language capability to generate professional article, it will be non-trivial to distinguish whether such a scientist is human or AI. If a developer of an AI Scientist determined to create a virtual persona of a scientist with an ORCID iD, for demonstration of technological achievement, product promotion, or for another motivation, it would be almost impossible to distinguish between the AI and human scientist. The challenge shall be considered practically achieved when the Nobel Prize committee is alerted for any confusion on potential recipients. We might expect an AI Scientist detection system to be developed to identify who is an AI Scientist or not, that may resemble Deckard’s interrogation of Rachael in Blade Runner.

It shall be made clear that this goal does not state or imply that “all major discoveries will be made by AI Scientists”, nor that completeness of hypotheses or discoveries made by AI Scientist will be achieved. The challenge is fundamentally different from any attempts to prove the completeness of the system, including the Hilbert Program intended to prove axiomatic completeness of mathematic where feasibility is debunked by Incompleteness theorems by Kurt Gödel. It simply implies: “among discoveries made by AI Scientists, there should be discoveries that are considered very significant at the level worthy of the Nobel Prize or beyond”. The challenge is initiated based on the belief any significant acceleration of scientific discovery would benefits our civilization. This will be achieved by creating an alternative form of scientific discovery, and will change the form of science as we know as well as uncovering the essence of scientific discovery. The utility of such technology shall benefit broader areas of science, industry, and society.

The core of the research program shall be about “Science of Science” rather than “Science of the process of science by human scientists”. As in the case of past AI grand challenges, the best and perhaps the only way to demonstrate that scientific discovery can be reformulated computationally is to develop an AI system that outperforms the best human scientists. Furthermore, it is not sufficient to have AI Scientist make one discovery, it shall generate a continuous flow of discoveries at scale. The fundamental purpose behind this challenge is to uncover and reformulate the process of scientific discovery and develop a scalable system to perform it that may result in an alternative form of scientific discovery that we have not seen before.

### Case studies: scientific discovery as a problem-solving

Herbert Simon argued that science is problem-solving in his article “The Scientist as Problem Solver”^18^. Scientists set themselves tasks of solving significant scientific problems. If this postulation holds, defining the problem and strategy and tactics to solve these problems is the essence of scientific discoveries.

An example of the discovery of cellular reprogramming leading to iPS cell and regenerative medicine by Shinya Yamanaka is consistent with this framework^19,20^. It has a well-defined goal with obvious scientific and medical implications, and search and optimization has been performed beautifully to discover cellular reprogramming capability using four transcription factors, now known to be Yamanaka factors^21^.

Another example is the discovery of conducting polymer by Hideki Shirakawa, Alan MacDiarmid, and Alan Heeger. It started with an accident that an intern researcher at Shirakawa’s Lab mistakenly used an abnormally high concentration of chemicals that formed thin film. Shikawaka noticed this accidental discovery and optimized the condition of thin-film formation. Then, with MacDiarmiad and Heeger, they identified a condition for conducting polymer formation. The initial experiments with an accidentally high dose of the chemical can be view as a stochastic search process where search space was extended beyond normal scope followed by extensive search and optimization for stable thin-film formation^22^.

**Fig. 1:**
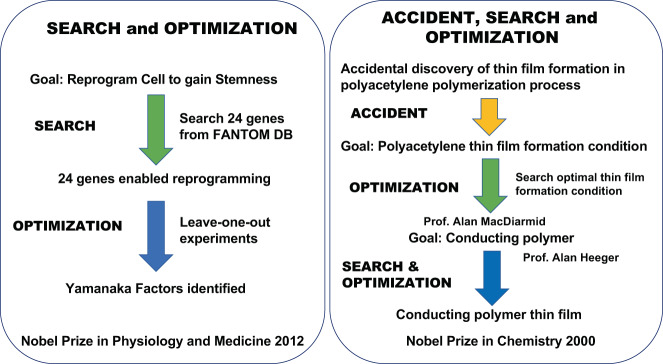
Very simplified process of scientific discoveries of iPS and conducting polymer. Search and optimization plays a critical role in the process of discovery. Yamanaka’s case is interesting because a search was conducted in bioinformatics followed by experiment-driven optimization that may be well suited for AI Scientist in the future.

The very simplified processes of these discoveries are shown in Fig. 1. These examples, among many other cases, exemplify the process of scientific discovery as problems solving and typical tactics are search and optimization.

### Deep and unbiased exploration of hypothesis space as an alternative form of science

Discovery with an exhaustive search of hypothesis space is what characterizes AI Scientist. In the traditional approach, scientists wish to maximize the probability that the discovery they make will be significant under certain criteria. In other words, scientists are focusing on making significant discoveries and are not interested in the number of discoveries made. This is the value-driven approach. In the alternative approach, the system will learn to maximize the probability that discovery at any level of significance can be made without imposing any value-driven criteria. This is an exploration-driven approach that is an unbiased exploration of hypotheses and makes sharp contrast against the current practice of science. This approach subsumes the problem-solving approach because specific problems will be included in hypotheses generated by an unbiased search of hypothesis space and their verification. This means AI Scientist will generate-and-verify as many hypotheses as possible, expecting some of them may lead to major discoveries by themselves or be a basis of major discoveries. A capability to generate hypotheses exhaustively and efficiently verify them is the core of the system and it can be the engine that gives horsepower to the system.

This transition of a value-based approach to exploration-based approach driven by the unbiased search of hypotheses space may resemble a transition from the intuition-driven design of experiments to unbiased exhaustive measurements represented by omics-approach made possible with microarray and high-throughput genome sequencers, combined with bioinformatics supported by powerful computing resources. Unbiased hypotheses generation and verification will be built on the unbiased measurement of the well-established omics-approach, hypothesis generation system, a series of machine learning and reasoning systems, and robotics-based experimental systems. This is a logical evolution of the modality of science where a vast hypothesis space is searched in an unbiased manner rather than depending on human intuition.

A potential argument against this approach is that such a brute force approach is too inefficient and may not lead to any significant discoveries. Furthermore, one may argue that asking the right question is most important in science rather than brute force exploration. It is interesting to note that in the early days of AI research, it was widely accepted that a brute force approach would not work for complex problems such as chess, and that heuristic programming was essential for very large and complex problems^23^. The actual history of AI clearly demonstrated massive computing and machine learning is the key to success as seen in DeepBlue^24^ and AlphaGo^25^.

Does that lessen apply to scientific discovery? There are three notable differences that are: (1) hypothesis space in scientific discovery is vast, open-ended, and possibly infinite as opposed to huge but finite space as in most games, (2) description in science, either knowledge or data, is not well-defined and often inaccurate whereas the well-defined description of game states and records exist in most games, and (3) evaluating hypothesis is likely to be more costly and time-consuming in science due to involvement of experiments. However, these issues can be made manageable and series of technologies to make them manageable will transform science and bring it to the next stage.

### The exploration of hypothesis space in scientific discovery

The hypothesis space for scientific discovery is huge and complex as opposed to very big but finite, well-defined, and monolithic state-space in games. State-space for games such as Chess, Shogi, and Go are finite, quantized, completely observable, and monolithic. For example, the game of Go is known to have a state-space complexity in order of 10^170^ and a game tree complexity in order of 10^360^. Every state of the game can be fully and unambiguously describable with a set of coordinates. There is no hierarchical structure in the state space. This is not the case in scientific discovery. The size of an entire hypothesis space is infinite or undefinable. States of objects involve substantial continuous values of higher-order dimensions. Nested hierarchical structures are prevalent. While it appears to be fundamentally different, much can be learned and adapted from experiences in building AI systems for gaming.

**Fig. 2:**
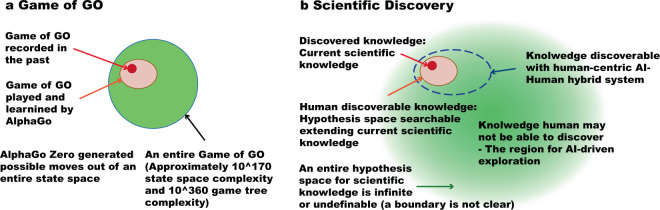
A possible space of exploration by AI Scientists. Search space structures for **a** perfect information games as represented by the Game of GO and **b** scientific discovery are illustrated with commonalities and differences. While the search space for the Game of GO is well-defined, the search space for scientific discovery is open-ended. A practical initial strategy is to augment search space based on current scientific knowledge with human-centric AI-Human Hybrid system. An extreme option is to set search space broadly into distant hypothesis spaces where AI Scientist may discover knowledge that was unlikely to be discovered by the human scientist.

The most recent and significant success of building AI system for a board game is AlphaGo series that beat the best human players. AlphaGo combined deep learning, reinforcement learning, and Monte-Carlo Tree Search (MCTS) to explore possible state space and game tree to learn best possible play within an explored state space^25^. State spaces are explored based on predictions of possible next moves generated by networks trained through supervised learning of past records of Game of Go (SL policy network) and MCTS expanded a search space. Reinforcement learning using self-play improves policy network and a value network is trained to properly evaluate game status. This approach enabled AlphaGo to learn how humans played, and how humans may play in the possible game state that has proximity to the past game (Fig. 2a, orange circle). AlphaGo Zero starts from a random move and learns to play purely using reinforcement learning without human knowledge^26^. Interestingly, AlphaGo Zero not only outperforms the best human players, it outperforms AlphaGo as well. This demonstrates the strength of unbiased exploration of state space as AlphaGo Zero explores an entire state space of Go where AlphaGo incrementally searches the vicinity of human play styles (Fig. 2a, green space). AlphaZero^27^ and MuZero^28^ further extend such approaches to be able to learn and exhibit superhuman capability in multiple different games by learning game dynamics with model-free and mode-based reinforcement learning, respectively.

A part of such an approach can be applied to scientific discovery. With AlphaGo levels of approach, a set of hypotheses can be generated using a body of knowledge accumulated to date, and it can be tested against a body of knowledge for their consistency and verified experimentally (Fig. 2b, orange circle). Enhancing the level of complexity of hypothesis and automation of experimental verification, exploration can be extended to hypothesis space where it was not practical with an incremental extension of current scientific practice (Fig. 2b, blue circle). The challenge would be to implement AlphaGo Zero strategy to randomly generate hypotheses for an entire hypothesis space because the hypothesis space can be infinite and undefinable (Fig. 2b, green zone). However, practical approaches may exist to solve this issue by leveraging the intrinsic structure of problem domains.

**Fig. 3:**
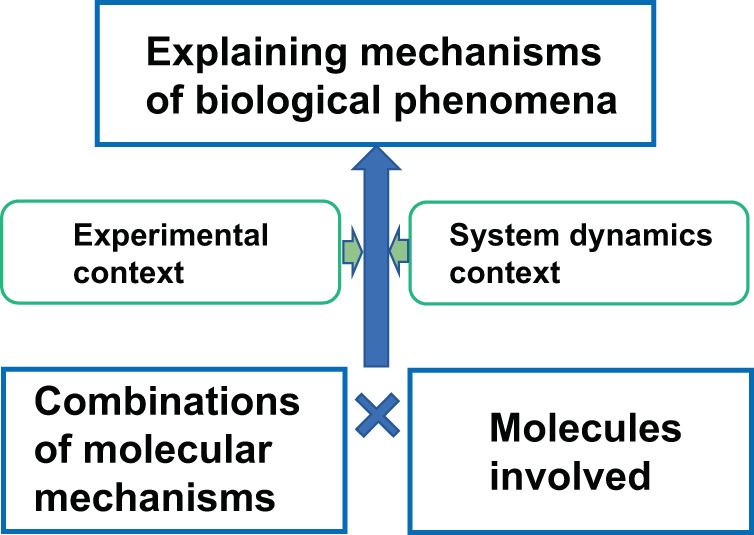
A basic structure of discovery in biology. Most discoveries in biology and medicine are concerned with the identification of mechanisms behind important biological processes. It can be fundamental processes such as cell cycle and cellular reprogramming or clinically relevant processes such as mechanisms of disease outbreak and progression. In many cases, this basic structure will be nested into multiple levels. It should be noted that “Molecular mechanisms” are biological processes by themselves, thus multi-layer construction of this basic structure of discovery are inevitable.

In biomedical sciences, any biological phenomena are the result of molecular interactions. It can be a simple interaction or involve a very complex network composed of very large numbers of molecules. Interactions among cells or even individuals can be attributed to molecular interactions. Information of any kind will be received by receptors to be meaningful for a biological system, therefore converted into molecular interactions. The exploration-driven approach in biomedical science leverages such intrinsic characteristics of application domains and may start from generating and testing hypotheses for basic biological mechanisms such as molecular interactions, genetic functions, metabolic reactions, material properties, and so forth and explore them at an unprecedented scale. Since most discoveries in biomedical science are on mechanisms behind diseases or specific biological phenomena exhaustive and unbiased exploration of molecular mechanisms shall be a building block for uncovering complex mechanisms for more complex biological phenomena (Fig. 3).

Therefore, it is reasonable to assume that the first stage of the project focuses on the hypothesis of a particular form, rather than unlimited and complex forms, specifically to identify molecular mechanisms behind biological processes. By focusing on the specific type of canonical form of knowledge, the problem is now relatively well-defined which is important as an opening game of the challenge. While the omics-approach uncovered massive data on genomes, transcriptomes, metabolomes, and interactomes, detailed and exhaustive characterization and precision measurements using low-throughput methods are required to verify specific molecular characteristics and nature interactions. Such processes are generally time-consuming and often not automated thus experiments are performed only for high priority targets. Automating such processes to match omics-scale enables exhaustive search and verification of a broader range of hypotheses, which shall lead to discoveries with high-impact biomedical and biotechnology applications.

There are pioneering works to turn this idea into reality. Adam, the first closed-loop system for scientific discovery, is designed to execute the discovery of orphan enzymes in budding yeast^11,13^. Eve was designed to perform an automated drug repositioning screen for neglected diseases and identified TP-470, originally developed as an angiogenesis inhibiting anti-cancer drug for its irreversible binding to methionine aminopeptidase-2, to be as effective as an anti-Malaria drug as a DHFR inhibitor^29^. These systems automated low-throughput assay processes and enabled exhaustive verification based on the hypothesis generated. These systems are highly automated, but not autonomous, as the problem to be solved and the process are fully designed by humans to the detail. These are special-purpose machines optimized for specific types of problems.

**Fig. 4:**
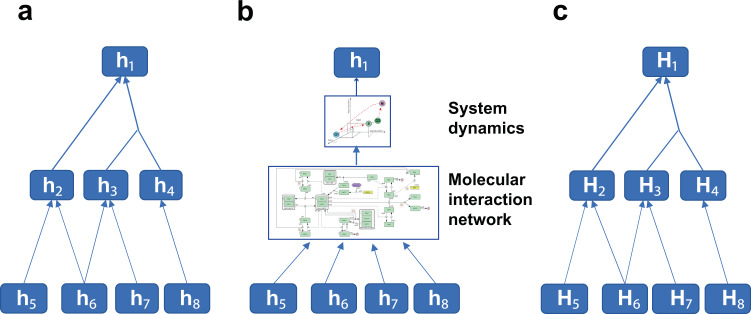
Hypothesis dependency tree. **a** Each hypothesis is dependent upon other hypotheses that are related to molecular mechanisms. **b** A hypothesis in question can be verified only at the system-level analysis of molecular interaction network behaviors, **c** a set of hypotheses and their dependency tree where each element is also a set (e.g., $H_1 = \left\{ {h_1^0,h_1^1,h_1^2, \cdots ,h_1^n} \right\}$). In massive and exhaustive search of hypothesis space, a set of hypotheses, rather than a single hypothesis, is generated to cover specific hypothesis space and verified.

This process can be applied iteratively (Fig. 4a). A biological process in questions (h_1_) may be explained by hypothesis h_2_ or a combination of h_3_ and h_4_, where h_2_, h_3_, and h_4_ may have possible underlying molecular mechanisms of h_5_ and h_6_, h_6_ and h_7_, and h_8_, respectively. In such a case, a process of hypothesis generation and verification will be performed iteratively to verify or reject h_1_ with a verified supporting mechanism either h_2_, h_3_, and h_4_. Generating experimental protocols and executing them is rather straightforward.

There are cases where a biological process can be only understood from a system dynamics perspective such as bifurcation and phase transition. A simple application of the iterative procedure to identify molecular mechanism is not sufficient. It requires reconstruction of molecular interaction network and analysis of their dynamical behaviors possibly underlying the process in question (Fig. 4b). This is more challenging as it requires the generation of hypothesis that link biological process with mathematical concepts and verifying them through experimental verification of network behaviors and molecular mechanisms composing the network.

**Fig. 5:**
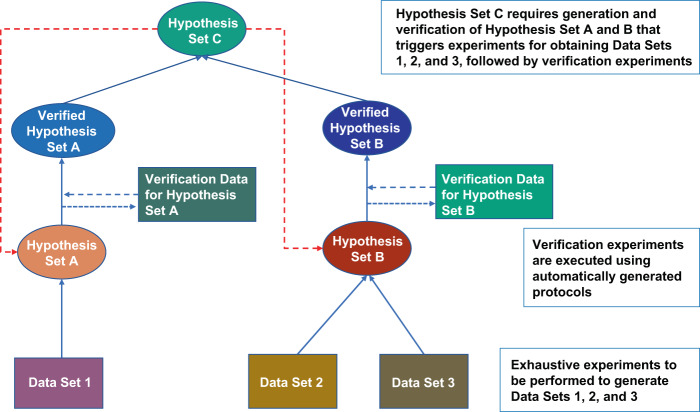
Hypothesis tree. A hierarchical generation of hypothesis sets and data to verify them will be automatically generated and executed. Verification of Hypothesis set C requires both Hypothesis sets A and B to be verified. Verification data for Hypothesis sets A and B shall be obtained from experiments in general. In general, multiple data sets are required to fill various parameters of elements in Hypothesis set before finally tested in the verification process. This requires Data Set 1 for Hypothesis set A, and Data Sets 2 and 3 for Hypothesis set B need to be collected. Data sets 1, 2, and 3 can be obtained from databases, or through automated experiments. Verified Hypothesis sets A and B mean a set of elements of Hypothesis sets A and B that are verified to be true or entire sets with a score for each element. Given the hypothesis set to be verified, this process automatically generates hypothesis sets that need to be verified first and specifies the data sets required.

Exhaustive exploration of hypothesis means a set of hypotheses is generated and verified rather than a single hypothesis. Thus, nodes of a hypothesis dependency tree in Fig. 4 shall be sets, rather than an element (Fig. 4c). Experiments shall be executed to verify an entire set of hypotheses, and protocols enabling such a process shall be generated. This may also include the generation of sub-hypothesis to be verified (Fig. 5).

**Fig. 6:**
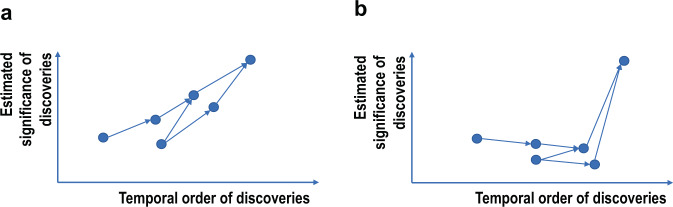
The landscape of estimated significance of the discovery. **a** The landscape is monotonic, and **b** the landscape is non-monotonic. A simplified illustration on why there are cases that research outcomes not immediately recognized to be significant lead to a major discovery. “Estimated significance of discoveries” is used only as a conceptual index. There is no rigid method to estimate the significance of the discovery. The numbers of citations and their temporal changes can be an interesting index, but it may be biased toward short-term popularity unless the time horizon is set appropriately.

The value of exhaustive generation of hypothesis and verification is in its potential capability to overcome the horizon problem. Assume that hypotheses are generated to maximize the expected significance of the discovery and tuned to focus highly expected value, such a strategy would work when the landscape is monotonically increasing (Fig. 6a). However, it may avert exploring paths to significant discovery, when a series of discoveries precondition to the significant one was low in expected significance (Fig. 6b). Searches may be terminated before reaching a significant discovery that may be located over-the-horizon. The landscape of discovery significance may be complex and non-monotonic. By enabling exhaustive exploration of hypothesis space, AI Scientist can go beyond the area that is over-the-horizon without it. At the same time, AI Scientist is not free from resource limitation. One of the most important areas of research would be to find out how to sample hypothesis space to effectively identify its landscape. Machine learning-guided experimental design was shown to be effective in chemistry^30^ and some of the principles can be applied here.

**Fig. 7:**
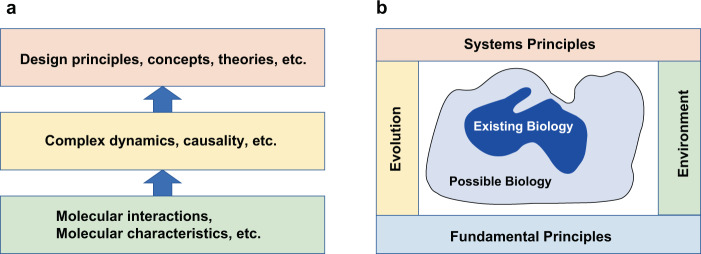
Layers of knowledge for unbiased exploration. **a** In scientific discovery, knowledge is layered from tangible knowledge to conceptual knowledge. Properties of molecules and their interactions are tangible and knowledge of systems dynamics and design principles are conceptual as they are not directly associated with tangible objects such as molecules and cells. Conceptual knowledge is often backed by mathematical and system-oriented theories. **b** Biology that we observe (“existing biology”) is constrained by multiple factors such as fundamental principles, systems principles, environmental constraints, and evolutionary selection. “Possible biology” meets all constraints but has not been observed or realized yet.

Knowledge of the world that science deals with is composed of multiple layers of abstraction, generally corresponding to the layers of systems in the domain. Discussions so far are centered around exploring and verifying hypothesis at molecular mechanisms, although it can be complex and nested. The next step shall be to uncover more complex phenomena and their dynamics that are interlinked with multiple layers of interaction, cells, organs, and individuals. This level further requires the identification of design principles and concepts behind complex systems (Fig. 7a). As discussed already, system dynamics play a central role in discoveries of this level, hence mathematical concept shall be linked to biological processes (Fig. 4b). At the same time, actual biological systems are constrained by fundamental principles such as biochemistry and physics, systems principles such as feedback theory and information theory, selected through evolution and manifested in the context of the environment it lives (Fig. 7b). AI Scientist need to learn what are possible and impossible and what possible biology exist at present, and this could be potentially similar to learning models of game dynamics^28^, but in open-ended and a highly complex environment.

The challenge is to find a way for the system to generate and test conceptual level hypotheses and test them. This shall be done in an unbiased manner. Methods such as model-based reinforcement learning and generative adversarial learning can be applied initially to investigate how to develop system that learn laws of nature at scale. Some recent studies demonstrate deep learning networks trained over millions of articles generate extensive molecular interactions^31^ and the potential relationship between molecules and disease only using articles a year (or years) before such a relationship was discovered^32^. Deep learning was also used to uncover hierarchical structure and functions of cells^33^, deep generative models for discovering hidden structures^34^, precision phenotyping to predict genetic anomalies^35^, and many more. The outcome of such approaches is a set of hypotheses generated by deep learning and other AI methods from unbiased data, and hypotheses are generated in an unbiased manner. Such predictions can be a basis for the search for in-depth molecular relationships and functions. Furthermore, recent success in the Ramanujan Machine^36^ in mathematics and the project Debater^37^ in adversarial reasoning augmented possible approach that can be incorporated in the hypothesis generation process. Qualitative physics offers the opportunity to generate, match, and explain physical and mathematical concepts such as bifurcation and phase transition^38,39^. Combined with the capability of deep learning neural network to learn, classify, and generate non-linear dynamics^40,41^, qualitative physics approach can be a powerful method for hypothesis generation and verification at the level of dynamical system concept. There are studies to use qualitative physics for biological processes^42,43^. This illustrates the potential of AI to be able to generate conceptual model exhaustively, assemble basic knowledge to be consistent with the conceptual model, and experimentally verify them. This approach essentially forces us to create a set of possible substructures of systems and search for structural matching with reality. With unbiased exploration at this level, AI Scientist shall be capable of exploring the complex dynamical system and may be able to discover new knowledge that is less likely to be discovered by human scientists.

### The multiverse of knowledge in scientific discovery

**Fig. 8:**
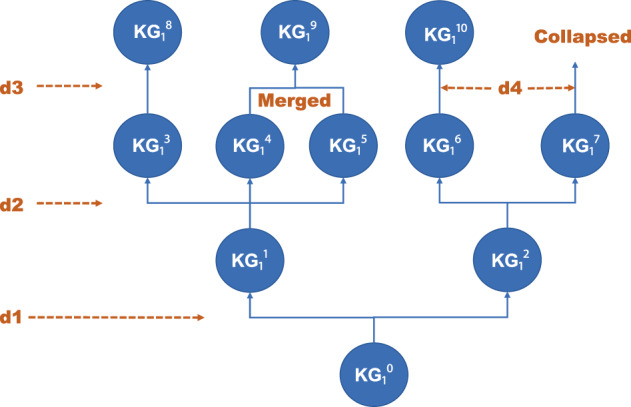
Evolving multiverse of knowledge graphs. The original knowledge graph (KG_1_^0^) is split into two incompatible KGs (KG_1_^1^ and KG_1_^2^) with the addition of a new data “d1” and associated arguments. Further addition of data “d2” resulted in the additional split of KGs. Data d3 and associated argumentation contextualized conflicting interpretations separating two KGs (KG_1_^4^ and KG_1_^5^) that resulted in the merger of them. Such a merge happens when KG_1_^4^ and KG_1_^5^ are not compatible due to conflicting interpretation of data d2, but data d3 and associated argumentation clarified conflict can be resolved that two interpretation of data d2 is context-dependent thus both interpretations hold in a different context. For two competing KGs (KG_1_^6^ and KG_1_^7^), d4 and associated argumentation eliminated KG_1_^7^, and KG_1_^6^ survived and augmented to be KG_1_^10^.

Generating hypotheses and maintaining a set of consistent body of knowledge in science is a formidable task due to the vast number of hypothesis generated and maintained, complexity and non-monotonicity, and unreliability of knowledge and data published. While publications and data already available today will be the initial foundation of hypothesis generation, the problem is that this initial foundation is not necessarily a solid ground; they contain substantial errors, missing information, and even fabrications. Manually checking statements with misinterpretation and biased interpretation of data individually and exhaustively is not practical given the volume of publications that shall be processed by AI Scientist. Currently, certain types of experimental results are known to be difficult to reproduce^44^, where some aspects of reproducibility issues shall be reduced by automated, transparent, and traceable experimental systems^45–47^. Intrinsic variability of biological systems due to noise and individual variations are treated as intrinsic features^48,49^ and shall be treated separately from ambiguities and inaccuracy caused by the process of research itself. Aside from the immediate reproducibility problem, some observations may hold true in some contexts but may not be applicable in the other context as an intrinsic nature of the complex system. Scientific knowledge is probabilistic and non-monotonic and a representation system shall be able to reflect this reality^50^. Knowledge shall be contextualized, and a new context can be added incrementally. While this is a nature of scientific research, this poses a serious issue in the computational process as hypothesis will be generated using a body of knowledge that is sure to be revised constantly. It is like making reasoning in the twilight zone where what is correct or not is always ambiguous. In this regard, hypothesis and knowledge cannot be clearly distinguished. It is a matter of degree of confidence. Verification in the context of inductive reasoning means that “a certain hypothesis is still surviving against all falsifiability challenges, thus considered most likely so far”. This implies for all hypotheses, survived or not, the trace of tests and their outcome need to be recorded. Unless errors are obvious, every statement in publications shall be converted into knowledge graph and the knowledge graph shall be constantly updated (Fig. 8). Obviously, inconsistencies will emerge which will trigger forks of knowledge graph, each of them consistent internally. Whenever some of the assumptions are altered, the relevant hypothesis shall be automatically re-evaluated. This can be accomplished by maintaining a very large number of multiple consistent set of knowledge and data with explicit breaking point which set to be considered more probable. Truth maintenance system, brief revision system, and non-monotonic reasoning can be applied to maintain consistency with multiple contexts^51–53^. Given the nature of scientific knowledge that is essentially probabilistic, multiple sets of knowledge graph shall be maintained persistently, unless sets are proven to be inconsistent, and likelihood of each knowledge set to be most probable change dynamically.

To evaluate the probability and possibly eliminate inconsistent knowledge sets, mechanisms to resolve ambiguities and falsify hypothesis need to be implemented. When a hypothesis is generated and verified, it always associated with data and justification why data support or reject the hypothesis. Theory of argumentation^54^ and non-monotonic reasoning shall be the basis of argumentation structure generation and processing^55,56^. Hypothesis, or claim, can be rejected or needs further delineation with multiple cases such as (a) data is fabricated, inaccurate, or incomplete, or (b) interpretation of data/assumption is not sufficient to justify the hypothesis, (c) scope of the hypothesis shall be limited, and (d) effective rebuttle exists that denies reasoning connecting data/assumptions and the claim. It is possible that reasoning presented in publications are insufficient to justify hypothesis, and detailed justification may need to be re-generated or argument against the claim to be created to make knowledge set complete. Recent progress in computational debate may be a first step to implement mechanisms to generate such argumentations^37,56,57^. The argumentation module shall generate argumentation to support or falsify existing hypothesis thereby justification can be strengthened through additional experiments and reasoning. At the same time, argumentation generated need to be understood by the human scientist. Qualitative simulation^38,58–60^ should be able to generate qualitative explanations consistent with human reasoning^39^. A closed-loop system involving such a process shall be developed that can incrementally improve confidence and consistency of knowledge and data thereby incrementally building up rigidly fortified data, argumentations, and hypotheses.

### Challenges in technology platform: automation, precision, and efficiency

Development of high precision, fast, and low operation cost experimental system and data analysis system is mandatory for this challenge. Unbiased search of hypothesis space means an unprecedented number of hypotheses will be generated and tested. The test requires both computational and experimental tests. The volume of experiments required to execute unbiased exploration would be a magnitude larger than current scientific practices. The revolutionary precise, cost-effective, and fast experimental systems need to be developed and deployed. Since the cycle of hypothesis generation and verification is the rate-limiting factor of the entire process, how fast and accurately perform experiments will determine the chance of success of the challenge. Some experiments will involve hypothesis exploring unusual conditions such as 1000 times off from the conventional parameters such as the concentration of chemicals. The first step would be to make laboratories fully connected and automated. Then, equipment will be replaced over time for high precision and efficient devices including microfluidics, followed by the use AI modules for each process before reaching the high level of autonomy expected in the AI Scientist.

Therefore, experimental systems shall be less resource-demanding and accurate yet reliable, reproducible, and integrated. While automation of various experimental processes has been commercialized already these are fragments of an entire process. The challenge requires an entire process of various types of experiments to be automated and part of such system may be installed as robotics cloud laboratory^46^. Recently, a robotics experimental system successfully identified proper condition for cell culture of medical-grade iPS-derived retinal pigment epithelial (RPE) cells after searching 200 million possible parameter combinations through Bayesian optimization with local penalization^47^. Optimization of lycopene biosynthesis pathway and biofuels for synthetic biology-based bio-manufacturing are other examples automated search of design space was shown to be effective^61,62^. Such success demonstrates the introduction of robotics-AI systems for each process shall improve the quality and efficiency of experiments. Automated closed-loop system impacts synthetic biology as well due to its quality and reproducibility^63^. Currently, only a system closed-the-loop of hypothesis generation and experimental verification is on budding yeast genetics^13^. Variety of experiments and their complexity shall be significantly augmented to cope with an extensive set of hypotheses to be verified. A literature analysis of over 1628 papers indicates 86–89% of experimental protocols in these papers can be automated by readily available commercial robotics systems^64^. This implies the progress can be quick initially, and what matters will be how to integrate different processes, data management, and how to automate processes that are not automated at this moment as well as novel protocols in future.

To achieve this, precise process management shall be imposed for the flows of control, materials, data, and physical agents. Due to vast numbers of experiments required for verification, experimental systems shall be compact and requirements for experimental samples and reagents shall be minimized. Organs-on-Chips is a recent addition to technologies that can reproduce experimental context closer to in vivo condition while maintaining controllability, traceability, and requires smaller amounts of experimental materials^65,66^. A novel origami-inspired surgical robot has interesting characteristics of being compact and high precision that can be applied for a range of experiments^67^. In future, the combination of microfluidics and robotics system will be used extensively in biological experiments to meet the demands of large numbers of experiments and requirements for controllability, accuracy, and traceability^68^.

Experimental devices shall be controlled by a platform that combines software tools, data access, and experimental systems embedded in the closed loop. Machine learning-guided experimental design was shown to be effective in chemistry^30^ and some of the principles can be applied to broader domains. Some of the technological platforms are readily available today, as seen in Garuda Connectivity and Automation Platform^69^, Wings workflow management tool^70,71^, and DISK Data Analysis and Hypothesis Evolution framework^72^, but many have to be developed as a part of the technology challenge. Extensive efforts are made to develop bioinformatics and systems biology analysis and modeling software and data standard that are fundamental to obtain data, analyze them properly, make accurate curation, and enabling dynamical simulations. Annual workshops such as COMBINE and HARMONY drive the development and adaptation of standards (http://co.mbine.org/home). Interoperability of software and data is mandatory to ensure connectivity of laboratory that is essential to automation of not only experimental processes but also analysis and modeling processes. More effort shall be made on the representation of hypothesis and knowledge reflecting the reality of scientific knowledge.

### Evolving relationship between AI Scientists, human scientists, and society

**Fig. 9:**
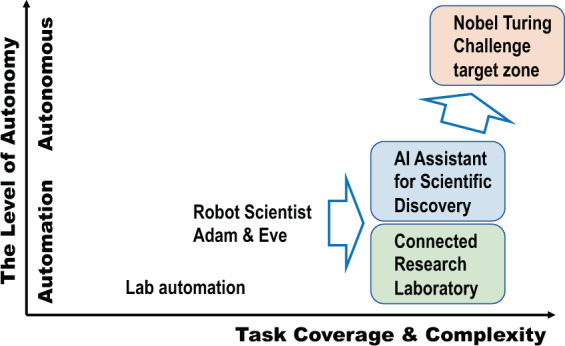
A possible path towards the Nobel Turing Challenge. AI Scientist requires a highly automated and connected laboratory to be able to design and execute experiments, as well as extensive access to databases and publication archives to process, extract, and evaluate current knowledge. Sophisticated laboratory automation is mandatory. Robot Scientist, Adam & Eve, is highly specialized automation with a certain level of intelligence for hypothesis generation and experimental protocol generation. The next step is to fully automate and connect laboratory equipment with layers of control for data flow, material flow, and physical control flow. Numbers of AI assistants shall be installed for each task initially, but need to be integrated as an integrated and highly autonomous system. The transition of automated system to autonomous system will be one of the most challenging part of the initiative.

How does AI Scientist evolve and transform scientific activities? It is clear superhuman-AI Scientist would not emerge out of blue. It will co-evolve with the scientific community over time. A possible, and logical, evolutional path of AI Scientist is to increase the level of automation first, followed by the increase of autonomy level (Fig. 9). Most current use of AI for research is a tool for specific tasks such as image classification, text-mining, and other tasks that are isolated and fully instructed by the human scientist. This is an AI tool stage. An early stage of AI scientist will take a form of a group of useful and highly competent software, including hypothesis generation module, and robotics to execute complex but pre-define tasks as instructed. Robot Scientist Adam and Eve are pioneering examples of this stage. Increasing repertoire of experiments and complexity of hypothesis are the next step. Substantial investment and user feedback are essential to make such systems useful and widely adapted.

Evolutionary pressure imposed on AI Scientist is whether it will be used by human scientists and widely adopted. Investment to develop AI Scientist, either by public funding or private sources, will be driven by the utility of such systems for human scientists. Therefore, AI Scientist will be inevitably interlocked with the research ecosystem of human scientists, and highly competent and user-friendly systems will survive for further development. This path inevitably make AI Scientist designed to be highly interactive with human scientists. Researchers will quickly understand the value and the power of AI Scientist, and will soon start asking questions that require the exhaustive generation of hypotheses and verification that exploits the full potential of AI Scientist at each stage of evolution. This will trigger the transformative change in biology as we witness in genomics when the unbiased measurement of genome sequence and transcriptome uncovered new realities in biology such as non-coding RNA^73,74^. Even without large-scale experiments, hypothesis generation capability of AI Scientist shall help researchers to explore hypotheses that may not be considered without such AI Scientist as well as being an extremely effective dialog-based creativity and discovery support system. Institutions without AI Scientist will no longer be competitive in science and technology.

With an increasing level of autonomy, AI Scientists are expected to make an autonomous decision on what to investigate next. While mechanisms to make it possible is yet to be seen, multiple strategies can be considered such as (1) goal-oriented approach of defining very high-level goals and find multiples paths to best achieve such goals or (2) bottom-up approach of exploring hypothesis search space based on discoveries already made by a specific AI Scientist. In either case, questions to be asked can be automatically extracted from publication, defined by human researchers, or randomly generating questions to be answered.

With an increased level of connectivity and flexibility to generate hypotheses and their verification process, instruction from human scientist will be more abstract, and AI Scientist will have an increased autonomous process to make decision of priorities of hypotheses to be tested and experimental protocols to be performed. This is a semi-autonomous stage because instruction on what to investigate is provided from outside although how to investigate them may be generated internally by AI Scientist. The level of abstraction of instruction by Human scientists need to be carefully chosen so that AI Scientist can execute the task with success. Instruction such as “find a set of protocols (transcription factors, chemicals, procedures) that can transform types of somatic cell (X) into defined cell types (Y)” is a difficult but tangible one. For such an instruction, multiple experimental protocol shall be generated, and prioritize choice of source and target cell types, interventions to use tested, and analysis procedures. However, much higher goals, such as “cure cancer”, “increase human life span to 150 years”, or “minimize climate change” would be problematic as some of these goals are too abstract, at least at the initial phase of AI Scientist. With the evolution of AI Scientist over years, some these questions may be addressed in future, still it requires user understanding on capability of AI Scientist to utilize its power.

With potentially expensive running cost of AI Scientists, especially when large-scale experiments are required, a certain level of monitoring will be enforced in most institutions. AI Scientist may include a function to generate questions more relevant to its owner or to the society. At least, it is highly plausible larger investment will be made to deliver high return on investment outcome. In this case, the choice of problem and evaluation of the significance of discoveries will reflect human-centric value system, most specifically the value of the stakeholders.

However, AI Scientists under this circumstance are less likely to make unexpected discoveries since the problems to be solved are pre-defined. Researchers with a priori expectations may sometime miss the big picture when one without such expectation may notice^75^. There are many cases discoveries initially received minor attention led to major discoveries later. It is extremely difficult, if not impossible, to evaluate the significance of the discovery when a few more discoveries may be needed to translate the discovery into high-impact outcome due to the over-the-horizon problem. The real value of AI Scientist is its capability to explore hypothesis space magnitude more efficiently into seemingly low-value domains with expectation that may eventually leads to major outcomes. Such systematic explorations into seemingly low-value hypothesis space are infeasible to be performed by human scientists. Both aspects of discovery are important that implies two roles for AI Scientist can be assumed that are “AI Scientist as a Problem Solver” aligned with the value of the stakeholders and “AI Scientist as an Explorer” that boldly explore hypothesis space nobody have gone before. However, in either cases, exhaustive hypothesis generation and verification will be the core of the AI Scientist that distinguishes it from the traditional approach.

**Fig. 10:**
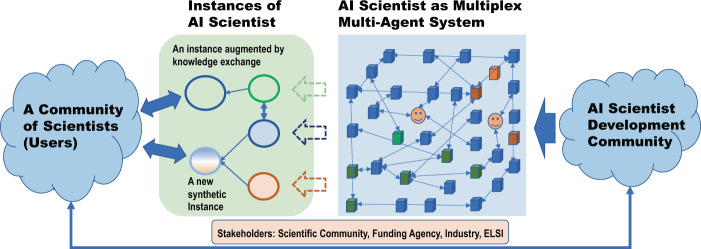
A possible configuration of AI Scientists: AI Scientist is a multiplexed multi-agent system where multiple instances of AI Scientist will be created. They evolve, merge, and interact with humans. Human experts can be a part of the system as human-in-the-loop system. Scientists who wish to work with AI Scientist are most likely to work with instances of AI Scientist.

AI Scientist will be a multiplexed multi-agent system generating multiple instances of AI Scientist (Fig. 10). It is comprised of many software and hardware agents (highly functional modules with a certain level of autonomy) with a high level of interactions, interoperability, and scalability in problem size and complexity. There may be two characteristics for an architecture of AI Scientist.

First, it may be a multiplexing multi-agent system. It is possible multiple instances of AI Scientist are created each specialized in a certain area extensively exploring hypothesis space organically. They are almost identical in components but differ in hypothesis space exploring. Communication among AI Scientists may enable them to merge discoveries for further exploration. This may take a form of communication between AI Scientists through a series of inquiries or the creation of a new synthetic new AI Scientist. Therefore, AI Scientist as a whole entails multiple instances of AI Scientist with focused areas. In this case, discoveries may be made systematically centered around initial core domains and eventually as a combination of multiple domains forming specific path-dependencies in discoveries. In the community of AI Scientist, a series of discoveries and publications made by AI Scientist may resemble that of the successful scientist. Interaction between AI Scientists is equivalent to the search and exchange of new knowledge and style of discovery specialized by each AI Scientist. When the critical mass of knowledge and data is required to generate significant hypothesis combining multiple domains, forming the community of AI Scientist would make sense. The discovery of CRISPR–Cas9 may be one of the examples of revolutionary discoveries coming from the combination of basic research seemingly distant areas of research^76^. It is well recognized that many discoveries considered groundbreaking was triggered by connecting two or more seemingly unrelated ideas. If AI Scientist shall be able to make discoveries of this nature, it must be able to access and connect very broad and less related domains where there is already a sufficient accumulation of knowledge and data by each AI Scientist.

Second, it may be a human-in-the-loop system. From AI Scientist perspective, agents composing them do not have to be exclusively software or hardware, it can be human expert as far as it can interface with the rest of the system. Human experts can be in the role of domain experts or in the commanding and monitoring role. The commanding and monitoring role is important to avoid misuse of the system. Potentially, AI Scientist can make discoveries that are harmful to human and our planet. What to discover fully depends on how the owner uses such capability. A strict ethical guideline and enforcement may be required with increased level of autonomy of AI Scientist. Ultimately, it will impact national security of the highest level.

There will be multiple AI Scientists either by institutions, academic community, country, or other societal boundaries. Some of them may communicate each other, some may be configured in isolation, and some would form local networks. Such configuration may be decided based on ownership of data and intellectual properties generated. Some modules and databases will be publicly maintained, and some would be proprietary. Although the level of autonomy can be very high, intellectual property is still retained with human researchers who run this system because human researchers make the decision to run AI Scientist and monitor their progress, and are responsible for the outcome. Some institutions may run AI Scientist at free-run modes with very high levels of autonomy, to let it explore hypothesis space that human researchers may not think of. Even in such a case, completely autonomous may not be achievable, as the intention of the owner will influence the running of the system.

### AI Scientist to transform systems biology and broader area of sciences

Automating the process of hypothesis generation and verification shall transform broad areas of sciences. Systems biology is one of the representative areas most affected by such technology, not only because it enables researchers to cope with massive data and publications otherwise under-utilized, but also it enables researchers to develop large-scale high-precision models as well as performing investigation significantly broader in scope and more extensively in parameter space than current approaches.

One of the initial expectations of systems biology was to develop a high-precision large-scale model of biological systems such as virtual humans that can be used as digital-twin of patients with in-depth molecular mechanisms behind^77,78^. While it is a holy grail of systems biology, it was proven to be extremely challenging as anticipated. There are fundamental difficulties for such a task partly due to the limitation of our cognitive capabilities and sociological constraints^14^. The research landscape of systems biology is clustered around two modalities that are; (1) a high-precision mechanistic model for the smaller and tractable system and (2) a large-scale network model based on omics data but less on detailed mechanisms. There is an inherent trade-off between these two modalities and attempts to overcome such trade-off have fallen short of expectations. First, there are human cognitive constraints. A vast amount of data and complexity of the system often goes beyond human comprehension and non-linear nature of biological process make things more difficult. Second, there are practical constraints as well. Building a large-scale precision model requires details of almost every interaction and molecular behavior to be investigated both computationally and experimentally which is beyond the capability of most research groups. Investigating each of such interactions and molecular behavior would require major efforts while many of them may not result in immediate major discoveries by itself. While interesting discoveries shall spring out from some of such efforts, tasks are designed to fill in every detail of a large model, rather than speculating the potential importance of interactions and molecules. It is not practical to assume dedicated efforts by members of the research group to be sustainable for many years unless most of such process is automated.

Perhaps, systems biology, particularly studies for large-scale precision models, is not a research field for human alone to investigate as possible causes of difficulties lies in human cognitive and sociological limitations. Once we accept the reality such a trade-off is inherent in human cognitive limitations and sociological constraints, the path to overcoming the trade-off is obvious. It is a field suitable for AI or AI-human hybrid system. Building high-precision large-scale models and efficiently exploit such models and aggregated knowledge to back it up requires powerful AI systems to support our scientific activities.

The AI system is not only useful for building large-scale in-depth models but will exhibit its power to discover new mechanisms and principles we have not imagined as well as discovering novel drug targets efficiently with a significantly extended search of target candidates. Extensive use of AI for drug discovery has been discussed with the implication of dramatically improving its efficiency and the transformation of the process^79^. Early successful cases including rapid identification of kinase inhibitor for DDR1 are encouraging^80^. A recent success of AlphaFold represents how AI technologies impact biomedical studies^81^. Studies on the relationship between drug target proteins and numbers of interactions of proteins demonstrate there is a low but reasonable probability that proteins with small numbers of identified interactions to be drug targets^82^. Although chances each protein can be a drug target may be small because the total numbers of such proteins are huge, exhaustive search of this class of proteins may result in abundant novel drug targets. With the same issues that arose in high-precision large-scale models, automation of the research process is essential to explore such opportunities.

Extending such an approach to synthetic biology to automate design and verification processes^63,83,84^.

Ultimately, a series of new discoveries will be integrated into an integrated model that is large-scale, high-precision, and in-depth. The implication is massive. It does not only mean researchers use AI Scientist as one of the tools, but it implies the practice of scientific discovery will be transformed dramatically with AI Scientist because discoveries will be made at scale and autonomously. At the same time, this will be a golden opportunity for systems biology since it will transform system biology into the next stage.

AI Scientist can be transformative not only in life science but also for broader science and technology domains. This is especially the case that requires hypothesis generation and verification to broader range parameter search of chemical synthesis and material discovery. Already, there are emerging interests in chemistry and material science for automation of experiments coupled with machine learning guide experimental design at various levels^30,46,85–90^. The idea of massive search of hypothesis space and verification applies to these domains as well. However, if such efforts can be applied to the discovery of novel concepts are yet to be seen. Recently, The Ramanujan Machine was announced for automated generation of conjectures in mathematics^36^. The Ramanujan Machine added a new perspective as it is not a parameter search and extensive generation of conjectures. With the rapid advances in robotics, sensors, AI with the increasing availability of computing powers, AI Scientists for broader domains of science will be inevitable. Research institutions without such capability will no longer be competitive in the coming decade.

The Nobel Turing Challenge is the ultimate challenge for AI and systems biology. Any progress toward achieving the goal will generate high utility technologies that shall accelerate science. Due to its breadth of expertise required and possible length of duration to achieve the goal, it may best be organized as a virtual big science^91^. Once the initiative taking off, it will uncover the essence of scientific discovery, and results in the creation of an alternative form of science. AI Scientist and human scientists will work together to solve formidable problems and to explore new intellectual territories where no one have gone before.
